# Numerical investigation of the impact of temperature-dependent thermal conductivity and viscosity on thermo-particle heat transfer through stationary sphere and using plume

**DOI:** 10.1371/journal.pone.0303981

**Published:** 2024-06-07

**Authors:** Hossam A. Nabwey, Muhammad Ashraf, Anwar Khan, Amir Abbas, A. M. Rashad, Zeinab M. Abdelrahman, Ehssan Ahmed Hassan, Mohamed M. Awad

**Affiliations:** 1 Department of Mathematics, College of Science and Humanities in Al-Kharj, Prince Sattam bin Abdulaziz University, Al-Kharj, Saudi Arabia; 2 Faculty of Engineering, Department of Basic Engineering Science, Menoufia University, Shebin El-Kom, Egypt; 3 Faculty of Science, Department of Mathematic, University of Sargodha, Sargodha, Pakistan; 4 Faculty of Science, Department of Mathematics, University of Gujrat, Sub-Campus, Mandi Bahauddin, Pakistan; 5 Faculty of Science, Department of Mathematics, Aswan University, Aswan, Egypt; 6 Department of Basic and Applied Sciences, College of Engineering and Technology, Arab Academy for Science & Technology and Maritime Transport (AASTMT), Aswan Branch, Aswan, Egypt; 7 College of Science and Humanities, Prince Sattam bin Abdul Aziz University, Al-Kharj, Saudi Arabia; 8 Faculty of Science, Suez Canal University, Ismailia, Egypt; 9 Faculty of Science, Department of Mathematics, Suez Canal University, El-Sheik Zayed, Ismailia, Egypt; College of Mathematics and Systems Science, Shandong University of Science and Technology, CHINA

## Abstract

Nanofluids have a wide range of applications due to their unique properties, such as enhanced thermal conductivity, convective heat transfer, and mass transfer. These applications can be seen in heat exchangers, cooling systems, and electronic devices to improve thermal performance. To enhance the cooling efficiency and lifespan of electronic devices such as smartphones, televisions, and computers nanofluids are used. These novel types of fluids can be used in energy storage systems, cancer treatment, imaging, and drug deliveryKeeping in mind, the real-time applications in engineering, industry, and science, the current study is carried out. In the present study for heat and mass transportation, the two-phase Buongiorno model for nanofluid is employed to scrutinize Brownian motion and thermophoresis aspects using stationary sphere and plume region. The temperature-dependent viscosity and thermal conductivity effects are encountered in momentum and energy equations, respectively are encountered. The proposed mechanism in the partial differential equations having dimensional form is converted to a non-dimensional form using appropriate dimensionless variables. The solution of the current non-linear and coupled model is obtained using the finite difference method. The numerical solutions presented in graphs and tables indicate that along with heat and mass transfer phenomena are entirely dependent on thermophoresis, Brownian motion, temperature-dependent viscosity, and thermal conductivity. The results indicate that the quantitative behavior of the velocity field is enhanced by increasing values of thermal conductivity variation parameters for both the sphere and the plume region at each position. On the other hand, the reverse trend is noted against the rising magnitudes of the viscosity variation parameter, thermophoresis parameter, and Brownian diffusion parameter. Additionally, the temperature in the plume region declines to enhance thermal conductivity variation parameter. A test for grid independence was performed by considering various grid points. Excellent solution accuracy has been seen as the number of grid points has risen. This ensures the validity and accuracy of the currently employed method. The current results are compared with already published solutions for the validation of the current model for specific cases. It has been noted that there is excellent agreement between both of the results. This close agreement between the results indicates the validation of the current solutions.

## 1. Introduction

Nanofluids as a combination of base fluid and a low concentration of nano-sized particles of metal or metal oxides are used in different fields of human activity, including engineering devices in power and chemical engineering, medicine, electronics, and others. The main reason for such a huge variety of nanofluid applications is the possibility, from one side, to enhance the heat and mass transfer due to the low concentration of nano-sized particles and, from the other side, to control the transport processes that can be used, e.g., in the drug delivery systems. Liquids with suspended nanoparticles have a higher thermal conductivity than standard liquids like water, methanol, and ethylene glycol. In many circumstances, fluids are essential heat-transfer media and play significant roles in a variety of industrial and engineering applications. The ability of a fluid to transfer heat, or thermal conductivity, is necessary for the efficient transfer of mass and energy. Given the importance of this property, researchers have dedicated a lot of effort and resources to improving the thermal conductivity of fluids. These efforts have led to the creation of a completely new class of fluids called nanofluids, which have a greater heat conductivity than traditional fluids. Nanofluids are created by dispersing nanoparticles in base fluids like methanol or water. The creation of nanofluids can be done in one or two steps, which is a significant advancement in fluid technology. Akoh et al. [1] proposed the one-step Vacuum Evaporation onto a Running Oil Substrate method, which entails a direct evaporation procedure. On the other hand, the method that first produces nanoparticles before dispersing them throughout the base fluids. To decrease particle buildup and disperse nanoparticles, ultrasonic devices are widely used. Masuda et al. in 1993 study [2] looked at how heat conductivity and liquid viscosity were affected by the dispersion of ultra-fine particles. Masuda et al.’s 1993 study [2] looked at how heat conductivity and liquid viscosity were affected by the dispersion of ultra-fine particles. For the first time, they altered the base fluid’s thermal conductivity using nanoparticles of AL_2 O_3, SiO_2, and TiO 2. Nanofluid is a novel class of fluid that was discovered in 1995 by Choi and Eastman [3]. They developed nanofluids that dispersed the nanoparticles throughout traditional base fluids, and they found that the thermal conductivities of these fluids were higher than those of ordinary fluids. Eastman et al. [4] and Lee et al. [5] both employed the two-step approach. By incorporating Al_2 O_3 into base fluids, they created a nanofluid. The two-step method is not as effective as the single-step method for metallic nanoparticles, but it is more efficient for oxide nanoparticles. One of two methods can be used to address the simulation challenges related to nanofluids: single-phase or two-phase models. In the single-phase model, these nanofluids are treated as pure fluids, therefore only the mass, momentum, and energy equations are applied. Obtainable from published theoretical and experimental correlations, effective thermal conductivity and viscosity, among other characteristics, are significant controls over nanofluids in this modeling technique. The thermal equilibrium and the no-slip condition between ultra-fine particles and base fluids are assumed by this modeling method. All of the fluids in this model have the same concentration of nanoparticles. Tiwari and Das [6] discussed the single model problem for nanofluids to show the heat transfer improvement in the two-sided lid-driven cavity heated and filled with nanofluids. The velocity slip in the two-phase model is not zero because of several factors like gravity. The investigations were carried out on the percentage of volume fraction of nanoparticles for better heat transfer augmentation [7]. Buongiorno in Ref. [8] established a two-phase mathematical model for heat transmission via convection mode in nanofluid. In this model, the influence of Brownian motion and thermophoresis has been emphasized. Researchers in various fields are actively exploring the application of nanofluids, aiming to exploit their potential.

Due to an increasing demand for energy conservation, rapid heat dissipation is critical in improving the efficiency of industrial operations, power production, electronics, and vehicle radiators. Researchers and engineers have tried multiple times to increase heat dissipation efficiency. Fluids such as ethylene glycol, oil, and water are insufficient to satisfy today’s demands due to their limited heat transmission capabilities. Nanofluids are a new class of high-potential working fluids that are employed in industry. Nanofluids are a colloidal combination of nanosized particles (less than 100 nm) in a standard heat transfer fluid that dissipates heat more efficiently than conventional fluids. Numerous studies have been done to look into the thermal and transport properties of nanofluids, and most of them have shown that nanofluids have a positive impact on heat dissipation. To predict numerical results in terms of local shear stress and local heat flux, Hussain et al. [9] investigated incompressible two-dimensional mixed convection flow of a viscous fluid down a vertical flat plate. Researchers in Refs. [10–14] thoroughly studied the many aspects of heat transfer phenomena in viscous fluid for different geometries. Duangthongsuk *et al*. [15] theoretically investigated nano particles with water as the basis fluid in the presence of temperature thermal conductivity and temperature-dependent viscosity. They analyzed the effects of the thermal conductivity and viscosity parameters in nanofluids and found that the heat transfer rate is increased in nanofluids as compared to the base fluid. Ding *et al*. [16] looked at the structural differences between nanoparticles and base fluids, as well as the relationship between thermal conductivity and shear stress viscosity. Eiyady *et al*. [17] investigated the boundary layer flow of water nanofluid and explored the effect of variable thermal conductivity and variable viscosity of water nanofluid on heat transfer enhancement in free convection. Rana *et al*. [18] have to use Finite Element Method (FEM) to investigate the effect of temperature dependent heat source/sink in mixed convection flow by taking water base nanofluids like *Cu*, *CuO*, *Al*_2_*O*_3_ and *TiO*_2_. Hady *et al*. [19] investigated the heat transfer properties and boundary layer fluid flow of a viscous nanofluid across a non-linear stretching sheet with thermal radiation and varied wall temperature. Elahi *et al*. [20] proceeded on to anticipate the analytical solution of Newtonian nanofluid flow in pipe and investigate the combined impact of MHD and temperature-dependent viscosity on heat transfer characteristics. The more relevant studies in nanofluids with distinct aspects and geometries are highlighted in ref. [21–23].

Due to the rapid development in nanotechnology, Mishra *et al*. [24] have given a brief review of the characteristics of nanofluid heat transfer taking into account viscosity as a function of temperature. Esfa *et al*. [25] investigated the influence of nanoparticle volume fraction on the thermal conductivity and dynamic viscosity of a water-hybrid nanofluid in an experimental study. Raju *et al*. [26] highlighted the effect of temperature-dependent and heat source / sink unsteady MHD nanofluid flow caused by rotating cone. Furthermore, they predicted that the heat transfer performance of *Ti*-water nanofluid is high when compared with Ti–alloy water nanofluid. Some other studies related to temperature-dependent thermal conductivity and viscosity can be found in [27–29]. The natural convection boundary layer flow of nanofluids around the sphere and in the plume region was examined by Ashraf et al. [30]. Khan et al. [31] investigated these processes as well as how heat generation impacted the free convection flow of magneto nanofluid around a sphere in the plume zone. A computational model of mixed convection fluid flow around a sphere was researched by Ashraf et al. [32], who also looked at the features of heat and fluid flow in the presence of thermophoretic transportation. The researchers carried out studies on the nano-fluids and hybrid nanofluids with various aspects and characteristics on several flow geometries given in [33–36]. Abbas et al. [37] also investigated the effects of temperature-dependent viscosity effects on thermophoretic motion force, and free convection boundary layer flow around the sphere. The combined effects of temperature-dependent viscosity and thermal conductivity on mixed convection flow along a magnetized vertical surface were discussed by Muhammad et al. [38]. Sparrow and Gregg [39] analyzed the phenomenon of free and forced convection flows over a flat plate for low Prandtl number values. Approximation of several heat and mass transfer problems along different shapes is given in [40–43].

By exploring the literature, it has been noted that in an enormous amount of work, nanofluid flow problems have been published. Scientists and researchers examined a lot, the heat transportation process in single-phase and two-phase models for nanofluid. However, a very large amount of work on the two-phase model (Buongiorno model) for nanofluid has been carried out due to the efficient carrier of heat transfer on the different geometries with distinct flow characteristics. But to the best of my knowledge nanofluid flow problems using a two-phase model (Buongiorno model) for nanofluid equipped with temperature-dependent viscosity and thermal conductivity past the stationary sphere and plume region have never been discussed before this work. The modeled problem in terms of coupled and non-linear partial differential equations is converted to dimensionless form, further, it is transformed to a primitive form and then solved using the finite difference method. The results for spherical and plume regions are plotted and tabulated under pertinent controlling parameters. The succeeding sections will elaborate on the molding, solution methodology, simulation process, and discussion of the results in detail.

## 2. Flow model and governing equations

**Fig 1:**
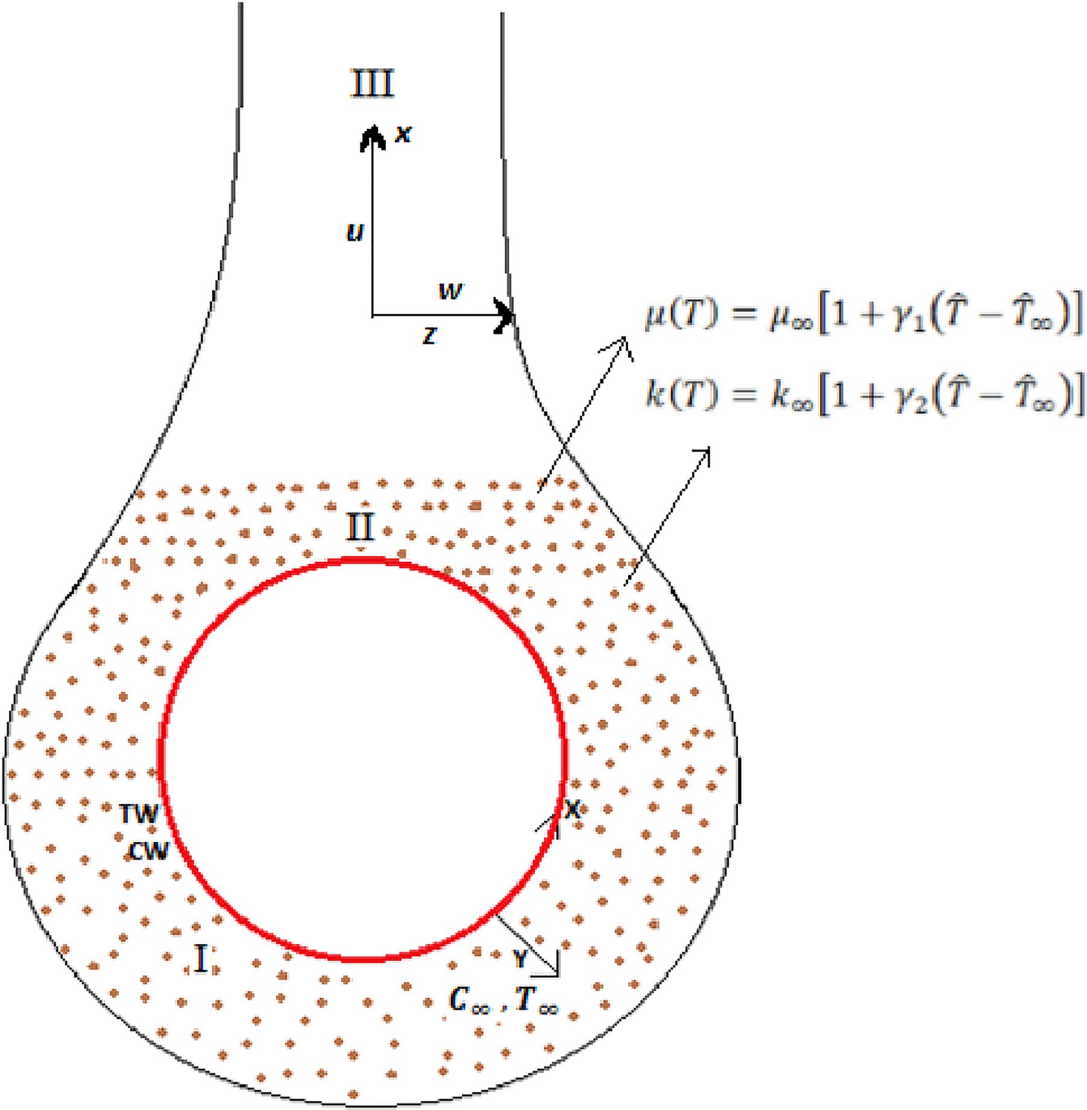
Flow configuration and coordinate system.

This section is confined to the modeling of the proposed problem with the following characteristics and assumptions:

- Two-dimensional, steady, and viscous flow of incompressible fluid past the sphere and plume region have been encountered.
- Two-phase model (Buongiorno model) for nanofluid with effects of Brownian diffusion and thermophoresis have been accomplished.
- Temperature-dependent viscosity and thermal conductivity are taken.
- The sphere of radius *a* as a flow geometry for natural convection is considered.
- The temperature of the surface of the sphere is taken as constant ${\hat{T}}_{w}$ with condition ${\hat{T}}_{w}>{\hat{T}}_{\infty }$, assuming ${\hat{T}}_{\infty }$ as the free stream temperature.
- The concentration at the surface of the sphere is taken as constant ${\hat{C}}_{w}$ with condition ${\hat{C}}_{w}>{\hat{C}}_{\infty }$, assuming ${\hat{C}}_{\infty }$ as the free stream concentration.
- The fluid flow configuration and coordinate system are demonstrated in Fig 1.
- The basic steady governing conservation equations for this fluid flow model can be written in Cartesian coordinates $\hat{x}$ and $\hat{y}$ as:

The structure of the fluid model is related to the geometrical configuration studied in [9,10,30–32].

The mass conservation equation;

$$\frac{\partial (\hat{r}\hat{u})}{\partial \hat{x}}+\frac{\partial (\hat{r}\hat{v})}{\partial \hat{y}}=0.$$ (1)

The momentum equation;

$$\hat{u}\frac{\partial \hat{u}}{\partial \hat{x}}+\hat{v}\frac{\partial \hat{u}}{\partial \hat{y}}=\frac{1}{\rho }\frac{\partial }{\partial \hat{y}}(\mu \frac{\partial \hat{u}}{\partial \hat{y}})+g\beta_{T}(\hat{T}-{\hat{T}}_{\infty })Sin\frac{\hat{x}}{a}+g\beta_{C}(\hat{C}-{\hat{C}}_{\infty })Sin\frac{\hat{x}}{a}.$$ (2)

The energy equation;

$$\hat{u}\frac{\partial \hat{T}}{\partial \hat{x}}+\hat{v}\frac{\partial \hat{T}}{\partial \hat{y}}=\frac{1}{\left(\rho C_{P}\right)}\frac{\partial }{\partial \hat{y}}(k\frac{\partial \hat{T}}{\partial \hat{y}})+\tau \{D_{B}\frac{\partial \hat{C}}{\partial \hat{y}}\frac{\partial \hat{T}}{\partial \hat{y}}+\frac{D_{T}}{{\hat{T}}_{\infty }}{\left(\frac{\partial \hat{T}}{\partial \hat{y}}\right)}^{2}\}.$$ (3)

The mass concentration equation;

$$\hat{u}\frac{\partial \hat{C}}{\partial \hat{x}}+\hat{v}\frac{\partial \hat{C}}{\partial \hat{y}}=D_{B}\frac{\partial^{2}\hat{C}}{\partial^{2}\hat{y}}+\frac{D_{T}}{{\hat{T}}_{\infty }}\frac{\partial^{2}\hat{T}}{\partial^{2}\hat{y}}.$$ (4)

Modeled boundary conditions;

$$\hat{u}=0=\hat{v},\;\hat{T}={\hat{T}}_{w},\;\hat{C}={\hat{C}}_{w}\;at\;\hat{y}=0,$$

$$\hat{u}\to 0=\hat{v},\;\hat{T}\to {\hat{T}}_{\infty },\;\hat{C}\to {\hat{C}}_{\infty }\;as\;\hat{y}\to \infty .$$ (5)

Where, the correlation formulae for temperature-dependent viscosity and thermal conductivity, respectively, used by [9,10] are given below:

$$\mu (T)=\mu_{\infty }\left[1+\gamma_{1}(\hat{T}-{\hat{T}}_{\infty })\right],$$

$$k(T)=k_{\infty }[1+\gamma_{2}(\hat{T}-{\hat{T}}_{\infty })].$$ (6)

Where, *μ*(*T*) and *k*(*T*) are temperature-dependent viscosity and temperature-dependent thermal conductivity, respectively. Here, $\varepsilon =({\hat{T}}_{w}-{\hat{T}}_{\infty })\gamma_{1}=\Delta T\gamma_{1}$ is viscosity variation parameter with *γ*_1_ as constant, and $\gamma =({\hat{T}}_{w}-{\hat{T}}_{\infty })\gamma_{2}=\Delta T\gamma_{2}$ is thermal conductivity variation parameter with *γ*_2_ as constant. Here, $\hat{u},\hat{v}$ denote the velocity components, parallel to the flow and vertical to the flow, respectively in dimensional form. The symbols *a* is the radius of the sphere, $\hat{r}$ is the dimensioned radial distance from the symmetric axis to the surface of a sphere, *β*_*T*_ is the thermal expansion coefficient, *ρ* is density of fluid, $\alpha =\frac{k_{\infty }}{\left(\rho C_{P}\right)}$ is thermal diffusivity, $\nu =\frac{\mu_{\infty }}{\rho }$ is the kinematic viscosity, *D*_*T*_ is thermophoresis coefficient, *g* acceleration due to gravity, *D*_*B*_ is Brownian diffusion coefficient, *β*_*C*_ is concentration expansion coefficient, *C*_*P*_ is specific heat at constant pressure, and $\tau =\frac{{\left(\rho C\right)}_{P}}{{\left(\rho C\right)}_{f}}$ is the ratio of nanoparticle heat capacity and the base fluid heat capacity. Notations *T*_*w*_, *C*_*w*_ are wall temperature and concentration, respectively. Notations *T*_∞_, *C*_∞_ are ambient temperature and concentration, respectively. Here, $\Delta T={\hat{T}}_{w}-{\hat{T}}_{\infty }$ is temperature difference between surface temperature and ambient temperature, *k*_∞_ is constant thermal conductivity, and *μ*_∞_ is constant dynamic viscosity.

## 3. Solution methodology

In this section, the solution methodology that is adopted currently for the solutions of the Eqs (1–4) along with boundary conditions given in Eq (5) is demonstrated in detail.

### 3.1. Nondimensionalization https://en.wikipedia.org/wiki/Nondimensionalization

To make the dimensionless, the partial differential equations given in Eqs (1–5), here, the following dimensionless variables used by [30] are introduced:

$$x=\frac{\hat{x}}{a},y={G_{r}}^{1/4}\frac{\hat{x}}{y},r=\frac{\hat{x}}{a},u={Gr}^{-1/2}\frac{a}{\nu }\hat{u},v={Gr}^{-\frac{1}{4}}\frac{a}{\nu }\hat{v},Gr_{T}=\frac{g\beta \Delta Ta^{3}}{\nu^{2}},Gr_{C}=\frac{g\beta \Delta Ca^{3}}{\nu^{2}},\theta =\frac{T-T_{\infty }}{T_{W}-T_{\infty }},\;\phi =\frac{C-C_{\infty }}{C_{W}-C_{\infty }}.$$ (7)

Using the above group of transformation, the dimensionless system of equations of the above fluid flow model is given below:

$$\frac{\partial (usinx)}{\partial x}+\frac{\partial (vsinx)}{\partial y}=0,$$ (8)

$$\frac{\partial u}{\partial x}+v\frac{\partial u}{\partial y}=(1+\varepsilon \theta )\frac{\partial^{2}u}{\partial^{2}y}+\varepsilon \frac{\partial \theta }{\partial y}\frac{\partial u}{\partial y}+\theta Sinx+\phi Sinx,$$ (9)

$$u\frac{\partial \theta }{\partial x}+v\frac{\partial \theta }{\partial y}=\frac{1}{Pr}\left[(1+\gamma \theta )\frac{\partial^{2}\theta }{\partial^{2}y}+\gamma {\left(\frac{\partial \theta }{\partial y}\right)}^{2}\right]+Nb\frac{\partial \phi }{\partial y}\frac{\partial \theta }{\partial y}+Nt{\left(\frac{\partial \theta }{\partial y}\right)}^{2},$$ (10)

$$u\frac{\partial \phi }{\partial x}+v\frac{\partial \phi }{\partial y}=\frac{1}{Sc}\left(\frac{\partial^{2}\phi }{\partial^{2}y}+\frac{Nt}{Nb}\frac{\partial^{2}\theta }{\partial^{2}y}\right).$$ (11)

With the designated boundary conditions;

$$\begin{array}{c} u=0,v=0,\theta =0,\;\phi =0,\;at\;y=0,\\ u\to 0,\theta \to 0,\phi \to 0,\;as\;y\to \infty .. \end{array}$$ (12)

Here, *u*, *v*, *θ*, and *ϕ* are non-dimensional velocity comments, temperature field and mass concentration, respectively.

In Table 1, The definitions of the parameters appeared in the Eqs (8–12) are given.

**Table 1:** Definitions of physical parameters.

$Pr=\frac{\nu }{\alpha }$	Prandtl number,
$Nb=\frac{{\left(\rho C\right)}_{P}D_{B}(C_{w}-C_{\infty })}{{\left(\rho C\right)}_{f}\nu }$	Brownian motion parameter
$Nt=\frac{{\left(\rho C\right)}_{P}D_{T}(T_{w}-T_{\infty })}{{\left(\rho C\right)}_{f}T_{\infty }\nu }$	Thermophoresis parameter
$\varepsilon =({\hat{T}}_{w}-{\hat{T}}_{\infty })\gamma_{1}=\Delta T\gamma_{1}$	Viscosity variation parameter
$Sc=\frac{\nu }{D_{B}}$	Schmidt number
$\gamma =({\hat{T}}_{w}-{\hat{T}}_{\infty })\gamma_{2}=\Delta T\gamma_{2}$	Thermal conductivity variation parameter

### 3.2. Primitive variable formulation

The dimensionless equations given in Eqs (8–11) along with boundary conditions given in Eq (12) are further transformed to a structure that is easy to put in the numerical algorithm of finite difference method for numerical solution in Fortran. The above-mentioned transformation is made using the following primitive variables used by [30] given below:

$$u(x,y)=x^{1/2}U(X,Y),v(x,y)=x^{-\frac{1}{4}}V(X,Y),Y=x^{-\frac{1}{4}}y,X=x,,\theta (x,y)=\theta (X,Y),\phi (x,y)=\phi (X,Y)$$ (13)

Using the Primitive Variable Formulation given in Eq (13), the system of partial differential equations provided in Eqs (8–11) with the specified conditions (12), is converted into a simple form for integration, and then the finite difference method is used to find the approximate solutions of the prescribed model using software Lahy Fortran -95. The transform form of the partial differential Eqs (8–11) with the specified boundary conditions given in Eq (12), are presented below by following [9,10,30–32].


$$X^{1/2}UcosX+\left\{X\frac{\partial U}{\partial X}-\frac{1}{4}Y\frac{\partial U}{\partial Y}+\frac{1}{2}U+\frac{\partial V}{\partial Y}\right\}sinX=0,$$ (14)

$$XU\frac{\partial U}{\partial X}+\frac{1}{2}U^{2}+\left(V-\frac{1}{4}YU\right)\frac{\partial U}{\partial Y}=(1+\varepsilon \theta )\frac{\partial^{2}U}{{\partial Y}^{2}}+\varepsilon \frac{\partial U}{\partial Y}\frac{\partial \theta }{\partial Y}-\theta sinX-\phi sinX,$$ (15)

$$XU\frac{\partial \theta }{\partial X}+\left(V-\frac{1}{4}YU\right)\frac{\partial \theta }{\partial Y}=\frac{\left(1+\gamma \theta \right)}{Pr}\frac{\partial^{2}\theta }{{\partial Y}^{2}}+\frac{\gamma }{Pr}{\left(\frac{\partial \theta }{\partial Y}\right)}^{2}+N_{b}\frac{\partial \phi }{\partial Y}\frac{\partial \theta }{\partial Y}+N_{t}{\left(\frac{\partial \theta }{\partial Y}\right)}^{2}$$ (16)

$$XU\frac{\partial \phi }{\partial X}+\left(V-\frac{1}{4}YU\right)\frac{\partial \bar{\varphi \phi }}{\partial Y}=\frac{1}{Sc}\left(\frac{\partial^{2}\phi }{\partial^{2}Y}+\frac{Nt}{Nb}\frac{\partial^{2}\theta }{\partial^{2}Y}\right)$$ (17)

With the designated transformed boundary conditions;

$$\begin{array}{c} U=0,V=0,\theta =1,\;\phi =1,\;at\;Y=0,\\ U\to 0,\theta \to 0,\phi \to 0,\;as\;Y\to \infty . \end{array}$$ (18)

### 3.3. Solution scheme

The transformed flow equations given in Eqs (14–71) with boundary conditions given in Eq (18) are solved using the solution scheme known as Finite Difference Method (FDM). The computational procedure adopted by [30,31] is applied with detailed explanation. The finite difference method is used and then the discretization equations are;

Discretized continuity equation:

$$V_{i,j}=\left\{V_{i-1,j}+\frac{1}{8}Y_{j}(U_{i+1,j}-U_{i-1,j})-\frac{1}{2}{\Delta YU}_{i,j}-X_{i}\frac{\Delta Y}{\Delta X}(U_{i,j}-U_{i,j-1})\right\}-\frac{cosX_{i}}{sinX_{i}}{{X_{i}}^{\frac{1}{2}}U}_{i,j}.$$ (19)

Discretized momentum equation:

$$\left((1+\varepsilon \theta_{ij})+[\frac{1}{2}\Delta Y(V_{i,j}-\frac{1}{4}Y_{j}U_{i,j})-\frac{\varepsilon }{4}(\theta_{i+1,j}-\theta_{i-1,j})]\right)U_{i-1,j}+({-\Delta Y}^{2}(\frac{1}{2}+\frac{X_{i}}{\Delta X})U_{i,j}-2\left(1+\varepsilon \theta_{ij}\right))U_{i,j}+(\left(1+\varepsilon \theta_{ij})-[\frac{1}{2}\Delta Y(V_{i,j}-\frac{1}{4}Y_{j}U_{i,j})-\frac{\varepsilon }{4}(\theta_{i+1,j}-\theta_{i-1,j})]\right)U_{i+1,j}={-X}_{i}U_{i,j}U_{i,j-1}\frac{{\Delta Y}^{2}}{\Delta X}+{\Delta Y}^{2}(\theta_{i,j}sinX_{i}+\phi_{i,j}sinX_{i}).$$ (20)

Discretized energy equation:

$$\left(\frac{1+\gamma \theta_{i,j}}{Pr}+[\frac{1}{2}\Delta Y(V_{i,j}-\frac{1}{4}Y_{j}U_{i,j})-(1+\gamma \theta_{i,j})+\gamma \frac{1}{4Pr}(\theta_{i+1,j}-\theta_{i-1,j})-Nb\frac{1}{4}(\phi_{i+1,j}-\phi_{i-1,j})-Nt\frac{1}{4}(\theta_{i+1,j}-\theta_{i-1,j})]\right)\theta_{i-1,j}+({-X}_{i}U_{i,j}\frac{{\Delta Y}^{2}}{\Delta X}+\frac{2(1+\gamma \theta_{i,j})}{Pr})\theta_{i,j}+\left(\frac{1+\gamma \theta_{i,j}}{Pr}-[\frac{1}{2}\Delta Y(V_{i,j}-\frac{1}{4}Y_{j}U_{i,j})-(1+\gamma \theta_{i,j})-\gamma \frac{1}{4P_{r}}(\theta_{i+1,j}-\theta_{i-1,j})-Nb\frac{1}{4}(\phi_{i+1,j}-\phi_{i-1,j})-Nt\frac{1}{4}(\theta_{i+1,j}-\theta_{i-1,j})]+\frac{1}{Pr}\right)\theta_{i-1,j}={-X}_{i}U_{i,j}\frac{{\Delta Y}^{2}}{\Delta X}\theta_{i,j-1}.$$ (21)

Discretized mass concentration equation:

$$\left([\frac{1}{2}\Delta Y(V_{i,j}-\frac{1}{4}Y_{j}U_{i,j})]+\frac{1}{Sc}\right)\phi_{i-1,j}+\left(-X_{i}U_{i,j}\frac{{\Delta Y}^{2}}{\Delta X}-\frac{2}{S_{c}}\right)\phi_{i,j}+\left(-[\frac{1}{2}\Delta Y(V_{i,j}-\frac{1}{4}Y_{j}U_{i,j})]+\frac{1}{Sc}\right)\phi_{i+1,j}=-\frac{1}{Sc}\frac{Nt}{Nb}(\theta_{i+1,j}-2\theta_{i,j}+\theta_{i-1,j})$$ (22)

Discretized boundary condition:

$$U_{i,j}=0=V_{i,j},\;\theta_{i,j}=0=\phi_{i,j}\;at\;Y_{i,j}=0,$$

$$U_{i,j}\to 0,V_{i,j}\to 0,\;\theta_{i,j}\to 0,\phi_{i,j}\to 0\;as\;Y_{i,j}\to \infty .$$ (23)

For accuracy of solution, convergence criterion for *U*, *V*, θ and ϕ variables is given below;

$$\max\left|U_{ij}\right|+\max\left|V_{ij}\right|+max|\theta_{ij}|\leq \epsilon ,max|\phi_{ij}|\leq \epsilon$$

Where, *ϵ* = 10^−5^. Here, in this simulation step size has taken as Δ*X* = 0.05 and Δ*Y* = 0.02. A computation is started from *X* = 0 to infinity. Here, Δ*X* and Δ*Y* are step sizes along *X* and *Y* axis, respectively.

## 4. Model validation and grid sensitivity test

In Table 2, a test for grid independency test has done by considering various grid points for Y = 10.0. Excellent solution accuracy has been seen as the number of grid points has risen, it has been noted. Additionally, the findings deviation for heat transfer rate, mass transfer rate and skin friction at position *X* = *π*/4 has been calculated from grid points 200 to 400. From the calculated results for skin friction, it is noted that the results deviation percentage is 0.0008%, for heat transfer rate is 0.005%, and for mass transfer rate is 0.004%. The usage of 200 grid points is adequate for the convergence and correctness of fallout calculated using the finite difference method, it may be inferred from the deviation of the computed results. Utilizing 200 grid points, the whole set of solutions for the current model is established. For this grid independent test the Prandtl number Pr = 7.0, Thermal conductivity variation parameter *γ* = 1.0, Schmidt number *Sc* = 10.0, temperature dependent viscosity variation parameter ε = 1.0, thermophoresis parameter *Nt* = 0.4, and Brownian motion parameter *Nb* = 0.2 at *X* = *π*/4.

**Table 2:** Mesh analysis or grid independence test for Pr = 7.0, *γ* = 1.0, *Sc* = 10.0, *ε* = 1.0, *Nt* = 0.4, and *Nb* = 0.2 at *X* = *π*/4.

No. of Grid points	${\left(\frac{\partial U}{\partial Y}\right)}_{Y=0}$	${-(\frac{\partial \theta }{\partial Y})}_{Y=0}$	${-(\frac{\partial \phi }{\partial Y})}_{Y=0}$
25.0	3.22837	0.97809	0.87809
50.0	3.33500	0.92792	0.87792
100.0	3.34338	0.92006	0.87315
125.0	3.34357	0.91955	0.87231
200.0	3.34358	0.91920	0.87120
250.0	3.34359	0.91916	0.87012
300.0	3.34361	0.91915	0.87001
350.0	3.34361	0.91915	0.87001
400.0	3.34361	0.91915	0.87001

In Table 3, the current results are compared with already published solutions for the validation of the current model for specific cases. It has been noted that there is excellent agreement between both of the results. This close agreement between the results indicate the validation of the current solutions.

**Table 3:** Validation of numerical model; comparison of ${\left(\frac{\partial U}{\partial Y}\right)}_{Y=0}$ with the results of Sparrow & Gregg [26], *γ* = 0.0, *Sc* = 0.0, *ε* = 0.0, *Nt* = 0.0, and *Nb* = 0.0 at *X* = *π*/2.

Pr	Sparrow & Gregg [39]	Present
0.03	0.93841	0.93740
0.02	0.95896	0.95870
0.008	0.99550	0.99400

## 5. Mathematical model formulation for the Plume Region-III

This section is restricted to the modeling of the proposed problem Plume Region-III with following characteristics assumptions:

- Two-dimensional, steady and viscous flow of incompressible fluid in plume region have been encountered.
- Two phase model (Buongiorno model) for nanofluid with effects of Brownian diffusion and thermophoresis have been accomplished.
- Temperature-dependent viscosity and thermal conductivity are taken.
- The wall temperature in the plume region is taken as constant ${\hat{T}}_{w}$ with condition ${\hat{T}}_{w}>{\hat{T}}_{\infty }$, assuming ${\hat{T}}_{\infty }$ as the free stream temperature.
- The wall concentration in the plume region is taken as constant ${\hat{C}}_{w}$ with condition ${\hat{C}}_{w}>{\hat{C}}_{\infty }$, assuming ${\hat{C}}_{\infty }$ as the free stream concentration.
- The fluid flow configuration and coordinate system are demonstrated in Fig 1.
- The basic steady governing conservation equations by following [9,10,30–32] for this fluid flow model can be written in Cartesian coordinates $\hat{x}$ and $\hat{z}$ as:

The continuity equation in Plume region-III:

$$\frac{\partial (\hat{z}\hat{u})}{\partial \hat{x}}+\frac{\partial (\hat{z}\hat{w})}{\partial \hat{z}}=0,$$ (24)

The momentum equation in Plume region-III:

$$\hat{u}\frac{\partial \hat{u}}{\partial \hat{x}}+\hat{w}\frac{\partial \hat{u}}{\partial \hat{z}}=\frac{1}{\rho }\frac{1}{\hat{z}}\frac{\partial }{\partial \hat{z}}\left(\mu (\hat{T})(\hat{z}\frac{\partial \hat{u}}{\partial \hat{z}})\right)-g\beta_{T}(\hat{T}-{\hat{T}}_{\infty })-g\beta_{C}(\hat{C}-{\hat{C}}_{\infty })$$ (25)

The energy equation in Plume region-III:

$$\hat{u}\frac{\partial \hat{T}}{\partial \hat{x}}+\hat{w}\frac{\partial \hat{T}}{\partial \hat{z}}=\frac{1}{\rho c_{p}}\frac{1}{\hat{z}}\frac{\partial }{\partial \bar{z}}\left(k(\hat{T})(\bar{z}\frac{\partial \hat{T}}{\partial \hat{z}})\right)+\tau \left\{D_{B}\frac{\partial \bar{C}}{\partial \hat{z}}\frac{\partial \hat{T}}{\partial \hat{z}}+\frac{D_{T}}{T_{\infty }}{\left(\frac{\partial \hat{T}}{\partial \hat{z}}\right)}^{2}\right\},$$ (26)

The concentration equation in Plume region-III:

$$\hat{u}\frac{\partial \hat{C}}{\partial \hat{x}}+\hat{w}\frac{\partial \hat{C}}{\partial \hat{z}}=D_{B}\frac{1}{\hat{z}}\frac{\partial }{\partial \hat{z}}\left(\hat{z}\frac{\partial \hat{C}}{\partial \hat{z}}\right)+\frac{D_{T}}{{\hat{T}}_{\infty }}\frac{1}{\hat{z}}\frac{\partial }{\partial \hat{z}}\left(\hat{z}\frac{\partial \hat{T}}{\partial \hat{z}}\right)$$ (27)

Modeled boundary conditions:

$$\hat{w}=0=\hat{u},\;\hat{T}={\hat{T}}_{w},\;\hat{C}={\hat{C}}_{w}\;at\;\hat{z}=0,$$

$$\hat{w}\to 0=\hat{u},\;\hat{T}\to {\hat{T}}_{\infty },\;\hat{C}\to {\hat{C}}_{\infty }\;as\;\hat{z}\to \infty .$$ (28)

Where, the correlation formulae for temperature-dependent viscosity and thermal conductivity, respectively, used by [9,10] are given below:

$$\mu (T)=\mu_{\infty }\left[1+\gamma_{1}(\hat{T}-{\hat{T}}_{\infty })\right],$$

$$k(T)=k_{\infty }\left[1+\gamma_{2}(\hat{T}-{\hat{T}}_{\infty })\right].$$ (29)

Where, *μ*(*T*) and *k*(*T*) are temperature-dependent viscosity and temperature-dependent thermal conductivity, respectively. Here, $\hat{u},\hat{w}$ denote the velocity components, parallel to the flow and vertical to the flow, respectively in dimensional form. Here, $\varepsilon =({\hat{T}}_{w}-{\hat{T}}_{\infty })\gamma_{1}=\Delta T\gamma_{1}$ is viscosity variation parameter with *γ*_1_ as constant, and $\gamma =({\hat{T}}_{w}-{\hat{T}}_{\infty })\gamma_{2}=\Delta T\gamma_{2}$ is thermal conductivity variation parameter with *γ*_2_ as constant. Here, *w*, *u* denote the velocity components, to the $\hat{z}$ axis and $\hat{x}$ axis. The symbols *a* is the radius of the sphere, $\hat{r}$ is the Dimensioned radial distance from the symmetric axis to the surface of a sphere, *β*_*T*_ is thermal expansion coefficient, *ρ* is density of fluid, $\alpha =\frac{k_{\infty }}{\left(\rho C_{P}\right)}$ is thermal diffusivity, $\nu =\frac{\mu_{\infty }}{\rho }$ is the kinematic viscosity, *D*_*T*_ is thermophoresis coefficient, *g* acceleration due to gravity, *D*_*B*_ is Brownian diffusion coefficient, *β*_*C*_ is concentration expansion coefficient, *C*_*P*_ is specific heat at constant pressure, and $\tau =\frac{{\left(\rho C\right)}_{P}}{{\left(\rho C\right)}_{f}}$ is the ratio of nanoparticle heat capacity and the base fluid heat capacity. Notations *T*_*w*_, *C*_*w*_ are wall temperature and concentration, respectively. Notations *T*_∞_, *C*_∞_ are ambient temperature and concentration, respectively. Here, $\Delta T={\hat{T}}_{w}-{\hat{T}}_{\infty }$ is temperature difference between surface temperature and ambient temperature, *k*_∞_ is constant thermal conductivity, and *μ*_∞_ is constant dynamic viscosity.

## 6. Solution methodology

In this section, the solution methodology that is adopted currently for the solutions of the Eqs (24–28) along with boundary conditions given in Eq (29) is demonstrated in detail.

### 6.1. Nondimensionalization https://en.wikipedia.org/wiki/Nondimensionalization

To make the dimensionless, the partial differential equations for Plume region-III, given in Eqs (24–27), here, the following dimensionless variables used in [30] are introduced:

$$x=\frac{\hat{x}}{a},z={G_{r}}^{1/4}\frac{\hat{x}}{z},r=\frac{\hat{z}}{a},u={Gr}^{-1/2}\frac{a}{\nu }\hat{u},w={Gr}^{-\frac{1}{4}}\frac{a}{\nu }\hat{w},Gr_{T}=\frac{g\beta \Delta Ta^{3}}{\nu^{2}},Gr_{C}=\frac{g\beta \Delta Ca^{3}}{\nu^{2}},\theta =\frac{T-T_{\infty }}{T_{W}-T_{\infty }},\;\phi =\frac{C-C_{\infty }}{C_{W}-C_{\infty }}.$$ (30)

Using the above-mentioned group of transformation given in Eq (30), the dimensionless system of equations of the above fluid flow model is given below:

$$\frac{\partial u}{\partial x}+\frac{\partial w}{\partial z}=0,$$ (31)

$$\frac{\partial u}{\partial x}+w\frac{\partial u}{\partial z}=\left[(1+\varepsilon \theta )\frac{\partial^{2}u}{\partial z^{2}}+\frac{\left(1+\varepsilon \theta \right)}{z}\frac{\partial u}{\partial z}+\varepsilon \frac{\partial u}{\partial z}\frac{\partial \theta }{\partial z}\right]-\theta -\phi ,$$ (32)

$$\frac{\partial \theta }{\partial x}+w\frac{\partial \theta }{\partial z}=\frac{1}{P_{r}}\left[(1+\gamma \theta )\frac{\partial^{2}\theta }{\partial z^{2}}+\frac{\left(1+\gamma \theta \right)}{z}\frac{\partial \theta }{\partial z}+\gamma {\left(\frac{\partial \theta }{\partial z}\right)}^{2}\right]+Nb\frac{\partial \varphi }{\partial z}\frac{\partial \theta }{\partial z}+Nt{\left(\frac{\partial \theta }{\partial z}\right)}^{2},$$ (33)

$$u\frac{\partial \varphi }{\partial x}+w\frac{\partial \varphi }{\partial z}=\frac{1}{S_{c}}\left(\frac{1}{z}\frac{\partial }{\partial z}(z\frac{\partial \varphi }{\partial z})+\frac{N_{t}}{N_{b}}\frac{1}{z}\frac{\partial }{\partial z}(z\frac{\partial \theta }{\partial z})\right).$$ (34)

The designated boundary conditions;

$$\begin{array}{c} w=0,u=0,\theta =0,\;\phi =0,\;at\;z=0,\\ w\to 0,\theta \to 0,\phi \to 0,\;as\;z\to \infty . \end{array}$$ (35)

Here, *u*, *w*, *θ*, and *ϕ* are non-dimensional velocity comments, temperature field and mass concentration, respectively.

In Table 4, The definitions of the parameters appeared in the Eqs (31–135) are given.

**Table 4:** Definitions of physical parameters appeared in the Plume region-III.

$Pr=\frac{\nu }{\alpha }$	Prandtl number,
$Nb=\frac{{\left(\rho C\right)}_{P}D_{B}(C_{w}-C_{\infty })}{{\left(\rho C\right)}_{f}\nu }$	Brownian motion parameter
$Nt=\frac{{\left(\rho C\right)}_{P}D_{T}(T_{w}-T_{\infty })}{{\left(\rho C\right)}_{f}T_{\infty }\nu }$	Thermophoresis parameter
$\varepsilon =({\hat{T}}_{w}-{\hat{T}}_{\infty })\gamma_{1}=\Delta T\gamma_{1}$	Viscosity variation parameter
$Sc=\frac{\nu }{D_{B}}$	Schmit number
$\gamma =({\hat{T}}_{w}-{\hat{T}}_{\infty })\gamma_{2}=\Delta T\gamma_{2}$	Thermal conductivity variation parameter

### 6.2. Primitive variable formulation

The dimensionless equations given in Eqs (31–34) along with boundary conditions given in Eq (35) are further transformed to a such structure that is easy to put them in numerical algorithm of finite difference method for numerical solution in Fortran. The above-mentioned transformation is made using the following primitive variables used by [30,31] given below:

$$\begin{array}{c} u=x^{\frac{1}{2}}U(X,Z),\;W=x^{-\frac{1}{4}}W(X,Z),\;x=X,Z=x^{-1/4}Z,\\ \theta =\theta (X,Z),\;\phi =\phi (X,Z) \end{array}$$ (36)

By using the variables given in Eqs (31–34) along with boundary conditions given in Eq (35)

$$Z\frac{\partial U}{\partial X}-\frac{Z^{2}}{4X}\frac{\partial U}{\partial Z}+\frac{3}{4}ZU+W+Z\frac{\partial W}{\partial Z}=0$$ (37)

$$U\frac{\partial U}{\partial X}+\frac{1}{2}U^{2}+\left(W-\frac{1}{4}ZU\right)\frac{\partial U}{\partial Z}=\left[(1+\varepsilon \theta )\frac{\partial^{2}U}{\partial Z^{2}}+\frac{\left(1+\varepsilon \theta \right)}{Z}\frac{\partial U}{\partial Z}+\varepsilon \frac{\partial U}{\partial Z}\frac{\partial \theta }{\partial Z}\right]-\theta -\phi$$ (38)

$$XU\frac{\partial \theta }{\partial X}+\left(W-\frac{1}{4}ZU\right)\frac{\partial \theta }{\partial Z}=\frac{1}{Pr}\left[(1+\gamma \theta )\frac{\partial^{2}\theta }{\partial Z^{2}}+\frac{\left(1+\gamma \theta \right)}{Z}\frac{\partial \theta }{\partial Z}+\gamma {\left(\frac{\partial \theta }{\partial Z}\right)}^{2}\right]+Nb\frac{\partial \bar{\varphi }}{\partial Z}\frac{\partial \theta }{\partial Z}+Nt{\left(\frac{\partial \theta }{\partial Z}\right)}^{2}$$ (39)

$$XU\frac{\partial \phi }{\partial X}+\left(W-\frac{1}{4}ZU\right)\frac{\partial \phi }{\partial Z}=\frac{1}{Sc}\left(\frac{1}{Z}\frac{\partial }{\partial Z}(Z\frac{\partial \phi }{\partial Z})+\frac{Nt}{Nb}\frac{1}{Z}\frac{\partial }{\partial Z}(Z\frac{\partial \theta }{\partial Z})\right)$$ (40)

Boundary conditions

W=U=0,ϕ=1,θ=1atZ=0,

W→0,ϕ→0,θ→0asZ→∞ (41)

The transformed model in primitive form is presented in Eqs (37–40) along with transformed boundary conditions given in Eq (41) is so smooth and has similar terms which makes it easy to code the algorithm.

### 6.3. Solution scheme

The transformed flow equations are given in Eqs (37–40) with boundary conditions given in Eq (41) are solved using the solution scheme known as the Finite Difference Method (FDM). The computational procedure adopted by [30,31] is applied with a detailed explanation. The finite difference method is used and then the discretization equations are;

Discretized continuity equation:

$$W_{i,j}=\frac{1}{\left(\Delta Z+Z_{j}\right)}\left\{Z_{j}W_{i-1,j}-Z_{j}(U_{i,j}-U_{i,j-1})\frac{\Delta Z}{\Delta X}+\frac{{Z_{j}}^{2}}{8X_{i}}(U_{i+1,j}-U_{i-1,j})-\frac{3}{4}{\Delta ZZ}_{j}U_{i,j}\right\}.$$ (42)

Discretized momentum equation:

$$\left\{[\frac{1}{2}\Delta Z(W_{i,j}-\frac{1}{4}Z_{j}U_{i,j})+(1+\varepsilon \theta_{i,j})\frac{\Delta Z}{{2Z}_{j}}-\frac{\varepsilon }{4}(\theta_{i+1,j}-\theta_{i-1})]+1+\varepsilon \theta_{i,j}\right\}U_{i-1,j}+\left\{-(X_{i}\frac{{\Delta Z}^{2}}{\Delta X}+\frac{1}{2}{\Delta Z}^{2})U_{i,j}-2(1+\varepsilon \theta_{i,j})\right\}U_{i,j}+\left\{-[\frac{1}{2}\Delta Z(W_{i,j}-\frac{1}{4}Z_{j}U_{i,j})+(1+\varepsilon \theta_{i,j})\frac{\Delta Z}{{2Z}_{j}}-\frac{\varepsilon }{4}(\theta_{i+1,j}-\theta_{i-1})]+1+\varepsilon \theta_{i,j}\right\}U_{i+1,j}={-X}_{i}U_{i,j}U_{i,j-1}\frac{{\Delta Z}^{2}}{\Delta X}+{\Delta Z}^{2}\left(\frac{X_{i}}{\Delta X}U_{i,j}U_{i,j-1}-\theta_{i,j}-\phi_{i,j}\right)$$ (43)

Discretized energy equation:

$$\left\{[\frac{1}{2}\Delta Z(W_{i,j}-\frac{1}{4}Z_{j}U_{i,j})+\frac{1+\gamma \theta_{i,j}}{Pr}\frac{\Delta Z}{{2Z}_{j}}-\frac{\gamma }{Pr}\frac{1}{4}(\theta_{i+1,j}-\theta_{i-1})-Nb\frac{1}{4}(\phi_{i+1,j}-\phi_{i-1,j})-Nt\frac{1}{4}(\theta_{i+1,j}-\theta_{i-1})]+\frac{1+\gamma \theta_{i,j}}{P_{r}}\right\}\theta_{i-1,j}+\left\{-\frac{{\Delta Z}^{2}}{\Delta X}X_{i}U_{i,j}-\frac{2(1+\gamma \theta_{i,j})}{Pr}\right\}\theta_{i,j}$$

$$\left\{-[\frac{1}{2}\Delta Z(W_{i,j}-\frac{1}{4}Z_{j}U_{i,j})+\frac{1+\gamma \theta_{i,j}}{Pr}\frac{\Delta Z}{{2Z}_{j}}-\frac{\gamma }{Pr}\frac{1}{4}(\theta_{i+1,j}-\theta_{i-1})-Nb\frac{1}{4}(\phi_{i+1,j}-\phi_{i-1,j})-Nt\frac{1}{4}(\theta_{i+1,j}-\theta_{i-1})]+\frac{1+\gamma \theta_{i,j}}{Pr}\right\}\theta_{i+1,j}=-\frac{{\Delta Z}^{2}}{\Delta X}U_{i,j}\theta_{i,j-1}$$ (44)

Discretized mass concentration equation:

$$\left\{[\frac{1}{2}\Delta Z(W_{i,j}-\frac{1}{4}Z_{j}U_{i,j})-\frac{\Delta Z}{{2Z}_{j}}]+\frac{1}{Sc}\right\}\phi_{i-1,j}+\left\{-X_{i}\frac{{\Delta Z}^{2}}{\Delta X}U_{i,j}-\frac{2}{L_{e}}\right\}\phi_{i,j}+\left\{-[\frac{1}{2}\Delta Z(W_{i,j}-\frac{1}{4}Z_{j}U_{i,j})-\frac{\Delta Z}{{2Z}_{j}}]+\frac{1}{Sc}\right\}\phi_{i+1,j}=-X_{i}U_{i,j}\frac{{\Delta Z}^{2}}{\Delta X}\theta_{i,j-1}+\frac{1}{Sc}\frac{Nt}{Nb}\left\{(1+\frac{\Delta Z}{{2Z}_{j}})\theta_{i+1,j}-(1-\frac{\Delta Z}{{2Z}_{j}})\theta_{i-1,j}-2\theta_{i,j}\right\}$$ (45)

Discretized boundary condition:

$$W_{i,j}=0=U_{i,j},\;\theta_{i,j}=0=\phi_{i,j}\;at\;Z_{i,j}=0,$$

$$W_{i,j}\to 0,V_{i,j}\to 0,\;\theta_{i,j}\to 0,\phi_{i,j}\to 0\;as\;Z_{i,j}\to \infty .$$ (46)

For accuracy of solution, convergence criterion for *W*,*U*,θ and ϕ variables is given below;

$$\max\left|W_{ij}\right|+\max\left|U_{ij}\right|+max|\theta_{ij}|\leq \epsilon ,max|\phi_{ij}|\leq \epsilon$$

Where, *ϵ* = 10^−5^, accuracy. Here, in this simulation step size has taken as Δ*X* = 0.05 and Δ*Y* = 0.02. A computation is started from *X* = 0 to infinity.

## 7. Result and discussion

This section discusses and concludes the behaviors of the velocity field (*U*), temperature field (θ), and concentration of nanoparticles (ϕ), and their gradients, which include heat transfer rate $\frac{\partial \theta }{\partial Y}$, rate of nanoparticle concentration $\frac{\partial \phi }{\partial Y}$, and skin friction $\frac{\partial U}{\partial Y}$, as well as the fluctuations of various flow parameters, under the pertinent parameters effects such as Prandtl number *Pr*, thermophoresis parameter *Nt*, Brownian motion parameter *Nb*, variable viscosity parameter *ε*, variable thermal conductivity parameter *γ*, and Schmidt number *Sc*. The numerical solutions for the governing characteristics under consideration are graphed and tabulated as well. The answer is divided into two sections: concerning the sphere and plume region-III.

### 7.1. Analysis of the material properties about the sphere

**Fig 2:**
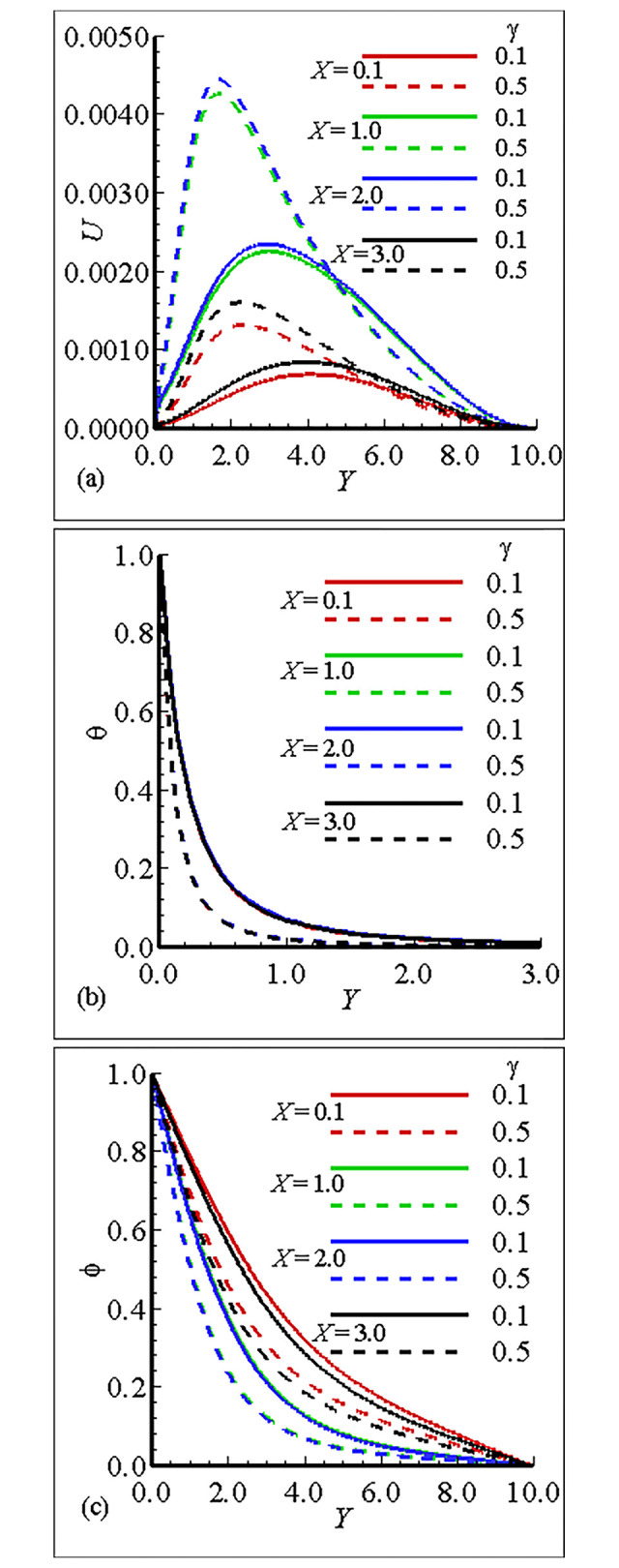
Variation of (a) *U* (b) θ and (c) ϕ for *γ* = 0.1, 0.5, when *ε* = 10.0, *Nb* = 0.4, *Nt* = 0.2, *Sc* = 0.5 and Pr = 7.0.

**Fig 3:**
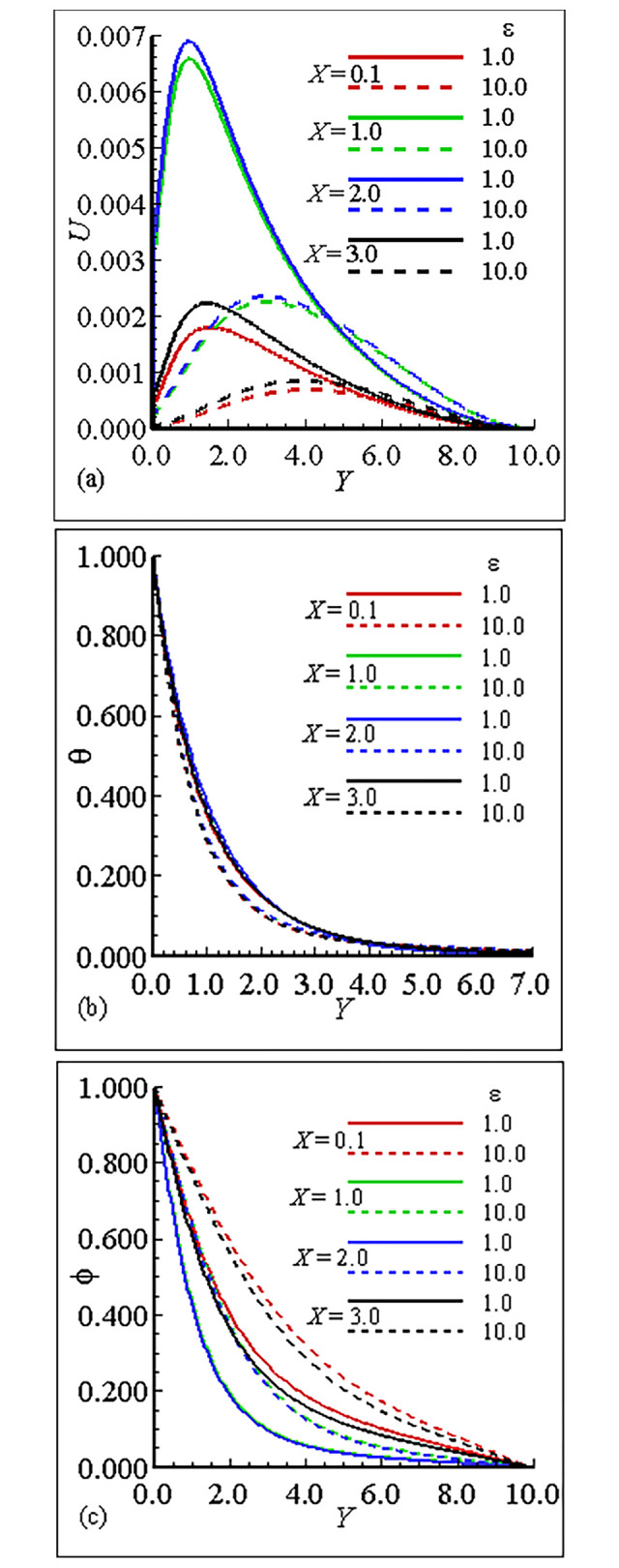
Variation of (a) *U* (b) θ and (c) ϕ for *ε* = 1.0, 10.0, when *γ* = 10.0, *Nb* = 0.4, *Nt* = 0.2, *Sc* = 0.5 and Pr = 7.0.

**Fig 4:**
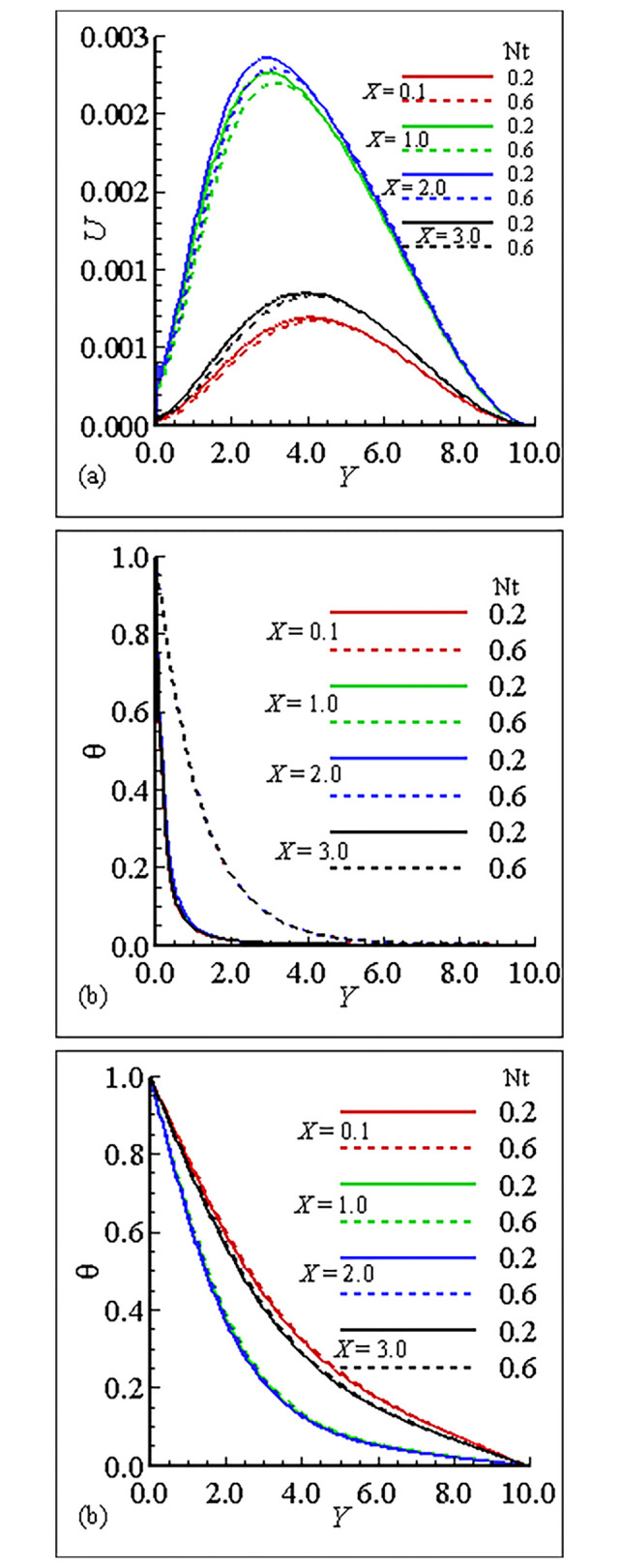
Variation of (a) *U* (b) θ and (c) ϕ for *Nt* = 0.2, 0.6, when *γ* = 10.0, *Nb* = 0.4, *Sc* = 0.5, ε = 1.0 and Pr = 7.0.

**Fig 5:**
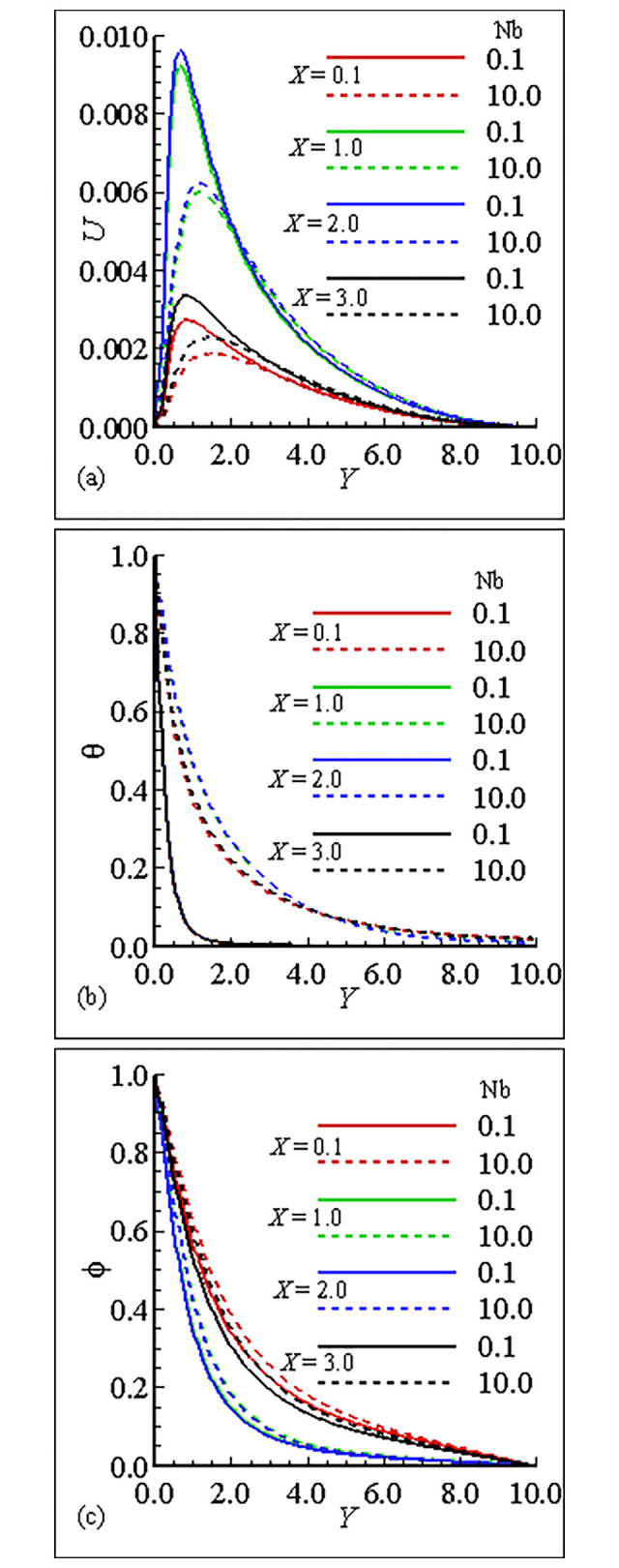
Variation of (a) *U* (b) θ and (c) ϕ for *Nb* = 0.1, 10.0, when *γ* = 10.0, *Nt* = 0.2, *Sc* = 0.5, *ε* = 1.0 and Pr = 7.0.

**Fig 6:**
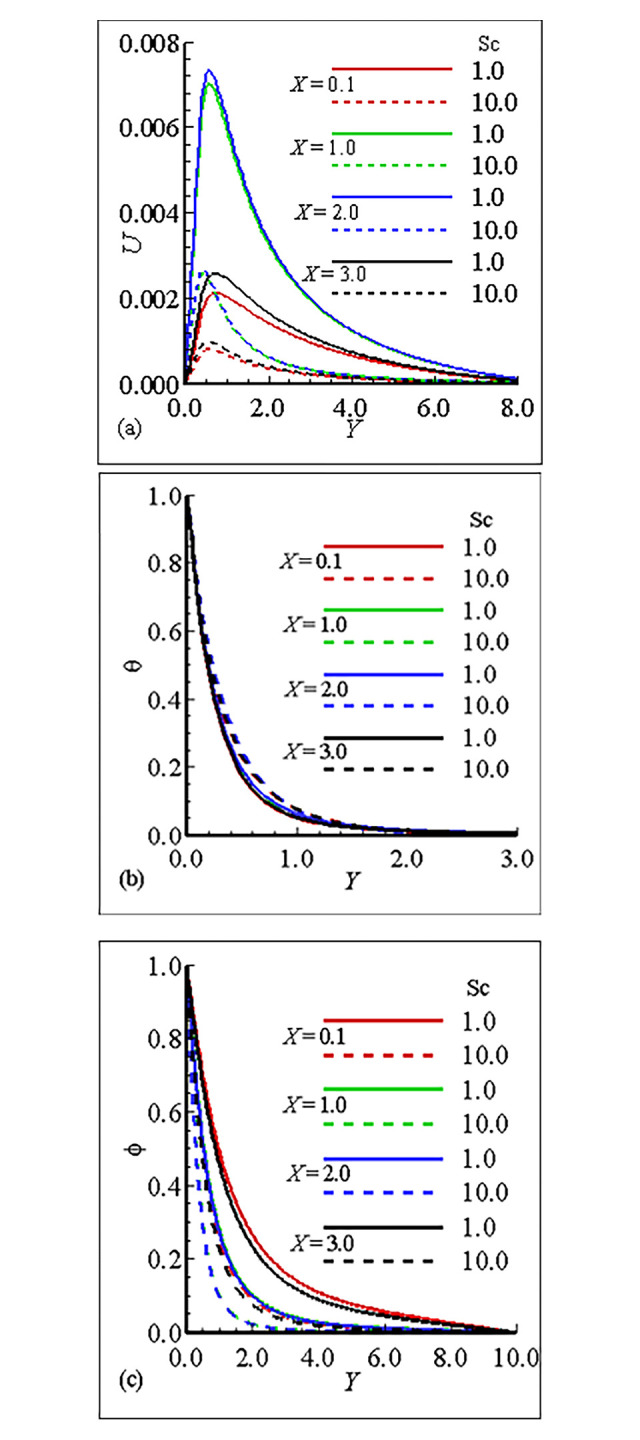
Variation of (a) *U* (b) θ and (c) *φ* for *Nb* = 0.1, 10.0, when *γ* = 10.0, *Nb* = 0.2, *Sc* = 0.5, *ε* = 1.0 and Pr = 7.0.

The interesting parameters in this study are the viscosity parameter *γ* and thermal conductivity parameter *ε*. The variation of these parameters on velocity (*U*) and temperature profile (*θ*), and nanoparticles concentration (ϕ) is given in Figs 2A–2C, 3A and 3B. It is interesting to notice that the quantitative behavior of *U* is maximum at position *X* = 3.0, and is minimum at position *X* = 0.1 and is increased but the temperature distribution and nanoparticles concentration are decreased for large values of *γ*. This is due to the increase in the frequency of intermolecular collision at higher temperature. Fig 3A–3C exhibit the effect of thermal conductivity variation parameter *ϵ*. From these figures we have learned that the velocity is maximum at position *X* = 2.0 for *ϵ* = 1.0 and the overall mechanism in this case is minimum for large values of *ε*. The temperature distribution for increasing values of *ϵ* and is uniform at each position. On the other hand the nanoparticles concentration in this case is maximum at position *X* = 0.1 and the nanoparticles concentration is decreased for increasing values of *ε*. Let us add some comments on the behavior of thermophoresis parameter *Nt* and *Nb* those are raised in mass equation during dimensionlization procedure and are plotted in Figs 4A–4C and 5A–5C. It is found that the *U* is greatest at position *X* = 2.0 and is lowest at position *X* = 0.1, however, temperature is increased for increasing values of *Nt* and is uniform at each position due the reason of uniformly heated sphere. Additionally, it is expected that, contrary to the behaviour of the velocity profile, the mass concentration will be at its highest point at position *X* = 0.1 and its lowest point at position *X* = 2.0. It is more instructive to plot, *θ* and ϕ versus Brownian motion parameter *Nb* as given in Fig 5A–5C. The computational results show that the *U* and *θ* are maximum for position at *X* = 2.0 and on the other hand ϕ is reduced at this position. The fluctuation of the Smith number Sc with respect to velocity, temperature distribution, and nanoparticle concentration is depicted in Fig 6A–6C. The velocity profile *U* reaches its maximum value at point *X* = 2.0 for *Sc* = 1.0 in the scenario when *Sc* is increased.With rising values of Sc, the temperature distribution *θ* increases significantly, whilst the concentration of nanoparticles decreases and reaches a lowest at position *X* = 2.0 for *Sc* = 10.0. Tables 5–7 exhibit the variation of thermal conductivity variation parameter *ϵ*, viscosity variation parameter *γ* and Brownian motion parameter *Nb*. In Table 5, it is predicted that the magnitude of skin friction is maximum at position *X* = 2.0 and is increased for large values of *γ*. It is also noted that the magnitude of heat transfer is maximum at position *X* = 0.1 and is increased with the increase of *γ* at each position. We also claim that the magnitude of mass transfer is maximum at point *X* = 0.1 and is increased by increasing *γ*. In this issue Table 6, exhibits the effect of various values of viscosity parameter *ϵ*. In this table we conclude that skin fraction and rate of mass transfer at point = 2.0, The rate of heat transfer is enhanced at point *X* = 1.0. Table 7 highlights the behavior for various value of Shmit Number *Sc*. As was expected the skin fraction and rate of mass transfer attains its maximum value at *X* = 2.0, and rate of heat transfer slightly reduced at this position.

**Table 5:** Variation of (a)Skin friction $\frac{\partial U}{\partial Y}$, (b) Heat Transer $\frac{\partial \theta }{\partial Y}$ and (c) Mass Transfer $\frac{\partial \phi }{\partial Y}$ for *ε* = 1.0, Pr = 7.0, *Sc* = 10.0, *Nt* = 10.0, and *Nb* = 1.0.

X	$\frac{\partial U}{\partial Y}$		$\frac{\partial \theta }{\partial Y}$		$\frac{\partial \phi }{\partial Y}$	
	*γ* = 1.0	*γ* = 10.0	*γ* = 1.0	*γ* = 10.0	*γ* = 1.0	*γ* = 10.0
0.1	0.00280	0.01805	9.27489	11.81390	0.11313	0.11681
1.0	0.02357	0.14780	9.27371	11.81352	0.11792	0.64341
2.0	0.02546	0.15931	9.27360	11.81349	0.11835	0.12578
3.0	0.00396	0.02548	9.27483	11.81388	0.11340	0.11728

**Table 6:** Variation of (a)Skin friction $\frac{\partial U}{\partial Y}$, (b)Heat Transfer $\frac{\partial \theta }{\partial Y}$ and (c) Mass Transfer $\frac{\partial \phi }{\partial Y}$ for *γ* = 1.0, Pr = 7.0, *Sc* = 10.0, *Nt* = 0.4, and *Nb* = 0.2.

X	$\frac{\partial U}{\partial Y}$		$\frac{\partial \theta }{\partial Y}$		$\frac{\partial \phi }{\partial Y}$	
	*ε* = 1.0	*ε* = 10.0	*ε* = 1.0	*ε* = 10.0	*ε* = 1.0	*ε* = 10.0
0.1	0.18352	0.01805	11.81388	11.81390	0.11708	0.11681
1.0	1.48084	0.14780	11.81342	11.81352	0.12714	0.12505
2.0	1.59420	0.15931	11.81337	11.81349	0.12802	1.21564
3.0	0.25875	0.02548	11.81386	11.81388	0.11766	0.11728

**Table 7:** Variation of (a)Skin friction $\frac{\partial U}{\partial Y}$, (b)Heat Transfer $\frac{\partial \theta }{\partial Y}$ and (c) Mass Transfer $\frac{\partial \phi }{\partial Y}$ for *γ* = 10.0, Pr = 7.0, *ε* = 1.0, *Nt* = 0.4, and *Nb* = 0.2.

X	$\frac{\partial U}{\partial Y}$		$\frac{\partial \theta }{\partial Y}$		$\frac{\partial \phi }{\partial Y}$	
	*Sc* = 0.1	*Sc* = 0.5	*Sc* = 0.1	*Sc* = 0.5	*Sc* = 0.1	*Sc* = 0.5
0.1	0.01812	0.01805	11.81390	11.81390	0.11588	0.11681
1.0	0.15241	0.14780	11.81386	11.81352	0.11761	0.12505
2.0	0.16466	0.15931	11.81386	11.81340	0.11776	0.12578
3.0	0.02562	0.02548	11.81388	11.81388	0.11598	0.11728

### 7.2. Physical behavior of material properties in the Plume Region-III

**Fig 7:**
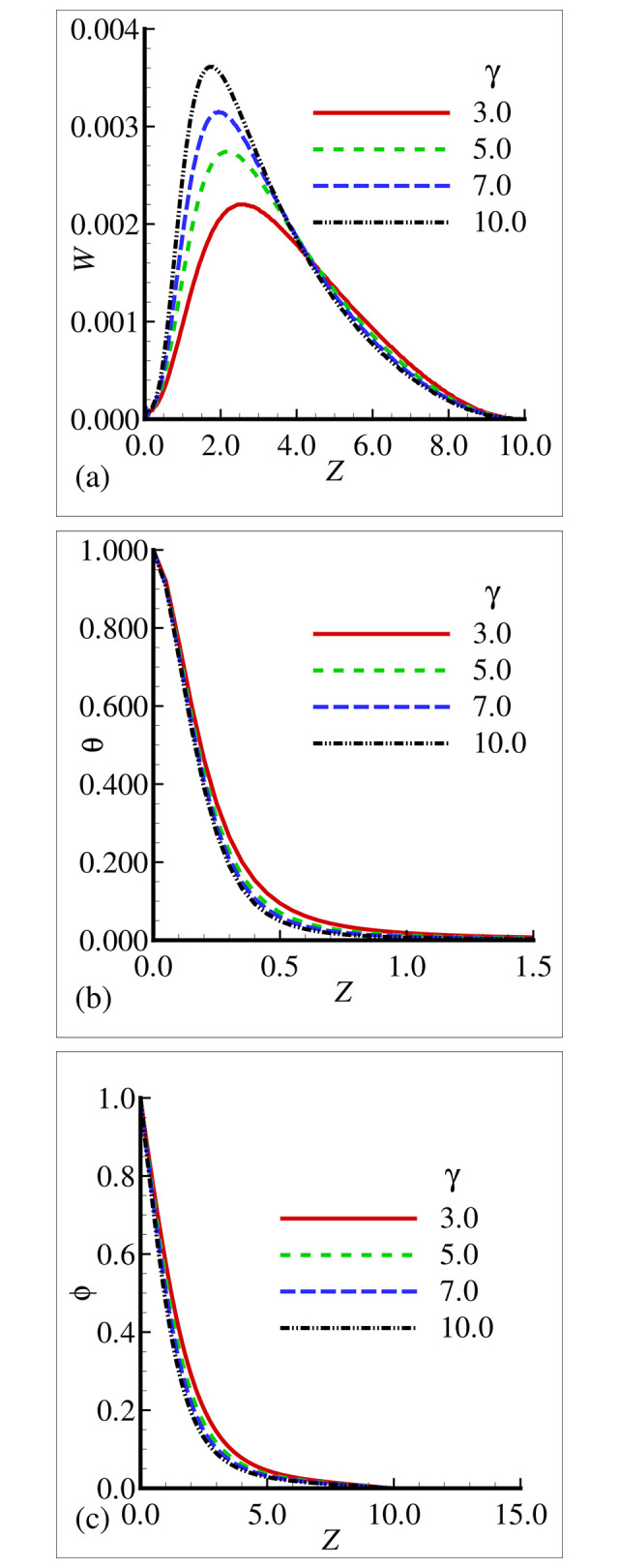
Variation of (a) *W* (b) θ and (c) ϕ for different value of *γ* when *ε* = 10.0, *Nb* = 0.4, *ε* = 10.0, *Sc* = 0.5 and Pr = 7.0.

This section is focused to examine the effects of involved flow parameters such as temperature dependent viscosity parameter *γ* and thermal conductivity variation *ε* on velocity distribution (*W*), temperature distribution (θ) and nanoparticles concentration (ϕ) as well as their gradients thatis $\frac{\partial W}{\partial Z},\frac{\partial \theta }{\partial Z}$ and $\frac{\partial \phi }{\partial Z}$ in the plume region-III. Also, the effects of thermophoresis parameter Nt, Brownian diffusion coefficient Nb, Prandtlnumber Pr, and Schmidt number Sc are included. The flow equations are solved by utilizing an efficient finite difference method and their solutions are portrayed in terms of graphs and tables. Fig 6A–6C are illustrating the physical behavior of *W*, θ and ϕ by integrating the effects of variable viscosity parameter *γ*, by keeping other parameters constant. Graphs are indicating that as *γ* is increased a rapid reduction in velocity profile is noticed but gross variations in temperature filed are observed. On the other side, in the mass concentration the rising trend is detected. Fig 7A–7C are exploring the influence of thermal conductivity variation parameter *ε* on *U*, θ and ϕ. Graphical findings are pointing that increasing values of *ε* result in decrements in velocity and temperature field but growing trend is noted in mass concentration. Table 8 expresses the variations in $\frac{\partial W}{\partial Z},\frac{\partial \theta }{\partial Z}$ and $\frac{\partial \phi }{\partial Z}$ corresponding to increasing change in viscosity variation parameter *γ*. From the Table 8 it is apparent that for four distinct values of the parameter *γ* display that reducing rate is yielded in velocity, temperature, and concentration derivatives. Table 9 describes the varying trend in $\frac{\partial W}{\partial Z},\frac{\partial \theta }{\partial Z}$ and $\frac{\partial \phi }{\partial Z}$ owing to increasing values of thermal conductivity variation parameter *ε*. It is also noticed from Table 9 that the accelerating tendency is perceived at the leading edge.

**Table 8:** Variation of (a)Skin friction $\frac{\partial W}{\partial Z}$, (b)Heat Transfer $\frac{\partial \theta }{\partial Z}$ and (c) Mass Transfer $\frac{\partial \phi }{\partial Z}$ for *γ* = 10.0, Pr = 7.0, *ε* = 1.0, *Nt* = 0.4, and *Nb* = 0.2.

*γ*	$\frac{\partial W}{\partial Z}$,	$\frac{\partial \theta }{\partial Z}$	$\frac{\partial \phi }{\partial Z}$
10.0	0.03301	1.90406	0.21772
20.0	0.01212	1.90403	0.20269
30.0	0.00727	1.90402	0.19325
40.0	0.00522	1.90400	0.18645

**Table 9:** Variation of (a) Skin friction $\frac{\partial W}{\partial Z}$, (b) Heat Transfer $\frac{\partial \theta }{\partial Z}$ and (c) Mass Transfer $\frac{\partial \phi }{\partial Z}$ for *γ* = 50.0, Pr = 7.0, *ε* = 10.0, *Nt* = 0.5, and *Nb* = 0.6.

*ε*	$\frac{\partial W}{\partial Z}$,	Percentage (%) Error	$\frac{\partial \theta }{\partial Z}$	Percentage (%) Error	$\frac{\partial \phi }{\partial Z}$	Percentage (%) Error
10.0	0.00355	5.3333 4.09207 4.86618	1.57346	10.27309 4.69459 3.59629	0.11081	1.81640 1.26848 1.35485
20.0	0.00375		1.75361		0.11286	
30.0	0.00391		1.83999		0.11431	
40.0	0.00411		1.90863		0.11588	

## 8. Conclusion

In the current proposed study, the mechanisms of heat and mass transfer in nanofluid flow past the sphere and in the plume region are performed. The two-phase model for nanofluid also known as the Buongiorno model is considered for convective heat and mass transfer. In this analysis of the Buongiorno nanofluid model empirical correlations for effective viscosity and thermal conductivity are encountered in which thermophoresis and Brownian diffusion are considered. The flow geometry is kept at a constant temperature. The effects of temperature-dependent viscosity and thermal conductivity on natural convection flow in nano-fluids via sphere and plume are the focus of the current investigation. The flow dimensionless and transformed flow equations are solved using the finite difference method(FDM). The results in graphs and tables for Region-I and region-III are computed and presented. The approximate solution that is valid over the entire domain has been numerically determined using FDM. The variation of viscosity parameter, thermal conductivity parameter, Brownian diffusion parameter, thermophoresis parameter, Schmidt number, and Prandtl number have been examined for various combinations of these parameters on velocity profile, heat transfer distribution, and nanoparticles concentration and their gradients solutions skin friction, rate of heat transfer, and other major physical parameters. The outcomes from these results are summarized below:

- The results indicate that quantitative behavior of velocity field is enhanced by increasing values of thermal conductivity variation parameter for both sphere and in plume region at each position. On the other hand, the reverse trend it noted against the rising magnitudes of viscosity variation parameter, thermophoresis parameter, Schmidt number, and Brownian diffusion parameter.
- The behavior of temperature profile is noted as in increasing trend for increasing values of thermophoresis parameter, Schmidt number, and Brownian diffusion parameter, but delineation is observed for increasing thermal conductivity variation parameter and viscosity variation parameter for sphere region. Additionally, temperature in plume region declines for enhancing thermal conductivity variation parameter.
- The trend in concentration profile notice as augment under the rising viscosity variation parameter, thermophoresis parameter, Brownian diffusion parameter and opposite attitude is seen for increasing Schmidt number and thermal conductivity variation parameter about sphere at it each position. In addition, the physical variable reflects decreasing trend in increasing thermal conductivity variation parameter in plume region.
- It can be noted that there is increasing behavior of skin friction for increasing values of thermal conductivity variation parameter and decreasing for increasing viscosity variation parameter and Schmidt number for spherical region. On the other side, the reverse tendency is perceived for same parametric conditions in plume region.
- It has been noted that the rate of heat transfer is rising for growing values of thermal conductivity variation parameter and viscosity variation parameter but declining for augmenting Schmidt number for spherical region but overall reverse plume region.
- The mass transfer rate shows that there is augmentation in it when thermal conductivity variation parameter and Schmidt number increase but a decrease is perceived for viscosity variation parameter in sphere and opposite for plume region.
- From the tabulated results, it is claimed that for the large value thermal conductivity parameter skin friction and rate of heat transfer is maximum and concentration of nano particle is reduced. Additionally, it can be shown that changes in the viscosity parameter had a significant impact on the fluid’s velocity, the rate of heat transfer, and the concentration of nanoparticles. Increasing the value of the viscosity parameter causes an increase in skin friction and the concentration of nanoparticles at each site.
- A test for grid independency test has done by considering various grid points and excellent solution accuracy has been seen as the number of grid points has risen. This ensures the method accuracy and validity.
- The current results are compared with already published solutions for the validation of the current model for specific cases. It has been noted that there is an excellent agreement between both of the results. This close agreement between the results indicates the validation of the current solutions.

### 8.1. Limitations of the current study

The limitations of the currently proposed study are given below:

- The current study is limited towards the nanofluid flow using two phase model for nanofluid (Buongiorno model) about the sphere and in the plume region.
- In the present study the empirical correlations for effective viscosity and thermal conductivity are encountered in which thermophsoresis and Brownian Diffusion are considered.
- In the current research temperature dependent viscosity and thermal conductivity effects are incorporated for the natural convection flow.
- The flow geometry is fixed and flow due to natural convection is assumed.
- In current study, for accuracy of solution, convergence criterion for *U*, *V*, θ and ϕ variables is given below;

$$\max\left|U_{ij}\right|+\max\left|V_{ij}\right|+\max\left|\theta_{ij}\right|\leq \epsilon ,\max\left|\phi_{ij}\right|\leq \epsilon .$$

Where, *ϵ* = 10^−5^. Here, in this simulation step size has taken as Δ*X* = 0.05 and Δ*Y* = 0.02. Here, Δ*X* and Δ*Y* are step sizes along *X* and *Y* axis, respectively. A computation is started from *X* = 0 to infinity.

- In the current investigations the ranges for the parameters are given as 0.1≤γ≤40,01≤ε≤40,0.2≤Nt≤0.6,0.1≤Nb≤10,0.1≤Sc≤10,0.008≤γ≤70.

### 8.2. Future recommendations

- This study can be enhanced to non-Newtonian fluid flow problem on the same geometry for different characteristics.
- This study is carried out for two phase model, this can be investigated for single phase model of nanofluids, hybrid nanofluids and ternary nanofluids.
- This in future can be extended to the impact of reduced gravity and shape factors of nanoparticles on non-Newtonian fluids flow problems using single phase model for nanofluid.
- This study can be carried for the hybridized nanofluid flow using multi-walled carbon nanotubes (MWCNTs) and single-walled carbon nanotubes (SWCNTs) as nano-particles with Water and ethylene glycol as bases fluids.

## Abbreviations

μ: Dynamic viscosity of nanofluid: Unit (*Pa*. *s*)
β_T_: Volumetric coefficient thermal expansion Unit (*K*^−1^)
($\hat{u},\hat{v},\hat{w}$): Dimensioned velocity components in horizontal and vertical axes and in $\hat{z}$ axis: Unit (*ms*^−1^)
θ: Dimensionless temperature
g: Gravitational acceleration Unit (*ms*^−2^)
*β*_*C*_(*K*^−1^): Volumetric coefficient concentration expansion
C: Mass concentration in boundary layer Unit (*kgm*^−3^)
v: Kinematic viscosity of nanofluid: Unit (*m*^2^*s*^−1^)
Nt: Thermophoresis parameter
(*u*,*v*,*w*): Dimensionless velocity components in horizontal and vertical axes and in z axis
*u*_∞_: Free stream velocity: Unit (*ms*^−1^)
(*U*,*V*,*W*): Dimensionless velocity components in horizontal and vertical axes and in Z axis in primitive form
Nb: Brownian motion parameter
ρ: Density of thefluid: Unit (*kgm*^−3^)
α: Thermal diffusivity of fluid: Unit (*m*^2^*s*^−1^)
ϵ: Thermal conductivity variation parameter
k: Thermal conductivity of the fluid Unit (*Wm*^−1^.*K*^−1^)
γ: Temperature dependent viscosity parameter
*a*(*m*): The radius of a sphere
C_p_: Specific heat of the fluid: Unit (*Jkg*^−1^.*K*^−1^)
C_f_: Skin friction coefficient
C_w_: Mass concentration in boundary layer at the surface Unit (*kgm*^−3^)
Nu: Nussle number
*C*_∞_: Mass concentration in free stream region Unit (*kgm*^−3^)
Sh: Sherwood number
$\hat{r}(m)$: Dimensioned radial distance from the symmetric axis to the surface of a sphere Unit (*m*)
Pr: Prandtl number
$\left(\hat{x},\hat{y},\hat{z}\right)$: Considered coordinates: Unit (*m*)
Gr_T_: Grashoff number
T: Temperature of the fluid: Unit (*K*)
T_W_: Temperature of the surface of Sphere Unit (*K*), Dimension [*K*]
Gr_C_: Modified Grashoff number
*T*_∞_: Temperature of free stream region Unit (*K*)
*k*_∞_: Thermal conductivity of the fluid at free stream region Unit (*Wm*^−1^.*K*^−1^)
D_B_: Brownian motion coefficient Unit (*m*^2^*s*^−1^)
∞: Ambient conditions
*μ*_∞_: Dynamic viscosity of nanofluid at frea stream region: Unit (*Pa*.*s*)
w: Wall conditions
